# Inhibition of NLRP3 inflammasome by MCC950 improves the metabolic outcome of islet transplantation by suppressing IL-1β and islet cellular death

**DOI:** 10.1038/s41598-020-74786-3

**Published:** 2020-10-21

**Authors:** Taisuke Matsuoka, Gumpei Yoshimatsu, Naoaki Sakata, Ryo Kawakami, Tomoko Tanaka, Teppei Yamada, Yoichiro Yoshida, Suguru Hasegawa, Shohta Kodama

**Affiliations:** 1 Department of Regenerative Medicine and Transplantation, Fukuoka University, 7-45-1 Nanakuma Jonan-ku, Fukuoka, 814-0180 Japan; 2 Department of Gastroenterological Surgery, Fukuoka University, Fukuoka, Japan

**Keywords:** Immunology, Endocrinology, Medical research

## Abstract

Early rejection is a critical issue to be overcome to achieve successful islet transplantation. NLRP3 inflammasome is a protein complex that mediates the maturation of pro-interleukin (IL)-1β and pro-IL-18 to IL-1β and IL-18, respectively, which induce cellular death. Here, we investigated the impact of NLRP3 inflammasome and the effect of its inhibition by MCC950 in a rodent model of islet transplantation. We assessed the therapeutic effects of MCC950, a specific inhibitor of NLRP3 inflammasome, on gene expression, islet survival ratio and viability, and islet transplantation in mice. NLRP3 inflammasome-related gene (*Nlrp3* and *Il1b*) expression was upregulated in islets stimulated with proinflammatory cytokines and suppressed when incubated with MCC950. Survival ratio and viability of incubated islets were reduced by cytokine stimulation and improved by MCC950. Regarding islet transplantation, the number of apoptotic cells in transplanted islets was reduced by MCC950. Furthermore, the expression of IL-1β in transplanted islets, migration of macrophages around islets, and fluctuation of blood glucose levels were suppressed by MCC950. Our study revealed that NLRP3 inflammasome worsened the therapeutic outcomes of islet transplantation and that MCC950 administration improved glycaemic control in syngeneic mice that underwent islet transplantation by inhibiting inflammation, which suppressed islet death.

## Introduction

Islet transplantation is a treatment option for insulin dependent diabetes mellitus that results from the autoimmune destruction of pancreatic beta cells. A phase 3 clinical trial, the Clinical Islet Transplantation Consortium Protocol 07 trial (CIT-07), reported that 87.5% of recipients achieved eradication of severe hypoglycaemic events with excellent glycaemic control at 1 year after the first transplantation with a median haemoglobin A1c level of 5.6%. However, multiple islet infusion is required to achieve a satisfactory outcome. Long-term islet graft survival in CIT-07/08 is now being followed up. Clinical islet transplantation is performed by infusion into the liver via the portal vein; however, a large number of islets are lost in the early phase of islet transplantation^1^. The major cause of the early rejection of islets is nonspecific innate immune responses represented by an instant blood-mediated inflammatory reaction (IBMIR). The activated innate immune system leads to a cytokine storm, involving tumor necrosis factor (TNF-α), interleukin-1β (IL-1β), and interferon-γ (IFN-γ)^2–5^. These cytokines activate macrophages immediately after the infusion of islets, and activated macrophages produce IL-1β, which accelerates the cytokine storm at the site of transplantation, causing a “negative chain” of events^6,7^. This series of inflammatory reactions causes severe damage to transplanted islets. Therefore, it is necessary to identify a strategy to regulate the nonspecific inflammatory response to promote the engraftment of islets and improve the therapeutic effect of islet transplantation.

NOD-, LRR- and pyrin domain-containing protein 3 (NLRP3) inflammasome is a protein complex in the cytoplasm that activates proinflammatory cytokines including IL-1β and IL-18 from pro-IL-1β and pro-IL-18, respectively. NLRP3 inflammasome comprises NLRP3 protein, an apoptosis-associated speck (ASC)-like protein and pro-caspase 1, which binds to the pyrin domain of NLRP3^8^. Once NLRP3 inflammasome is activated by signalling through Toll-like receptors, caspase-1 is activated and cleaves pro-IL-1β to IL-1β^9–11^. Recent studies reported that NLRP3 inflammasome contributed to β cell dysfunction and death in type 1 and 2 diabetes^7,12–14^. Furthermore, it affected the therapeutic effect of allogeneic organ transplantation^10^ and worsened bacterial/viral infections^11^. Although the regulation of NLRP3 inflammasome is a critical factor for the improvement of islet transplantation, the impact of NLRP3 inflammasome on islet transplantation is unclear.

MCC950, a specific inhibitor of NLRP3 inflammasome was previously shown to regulate various inflammatory diseases that correlated with IL-1β^15,16^. In this study, we investigated the impact and regulation of NLRP3 inflammasome on islet transplantation using a rodent model.

## Results

### NLRP3 inflammasome-related genes in cultured islets are upregulated by proinflammatory cytokines and suppressed by MCC950

**Figure 1:**
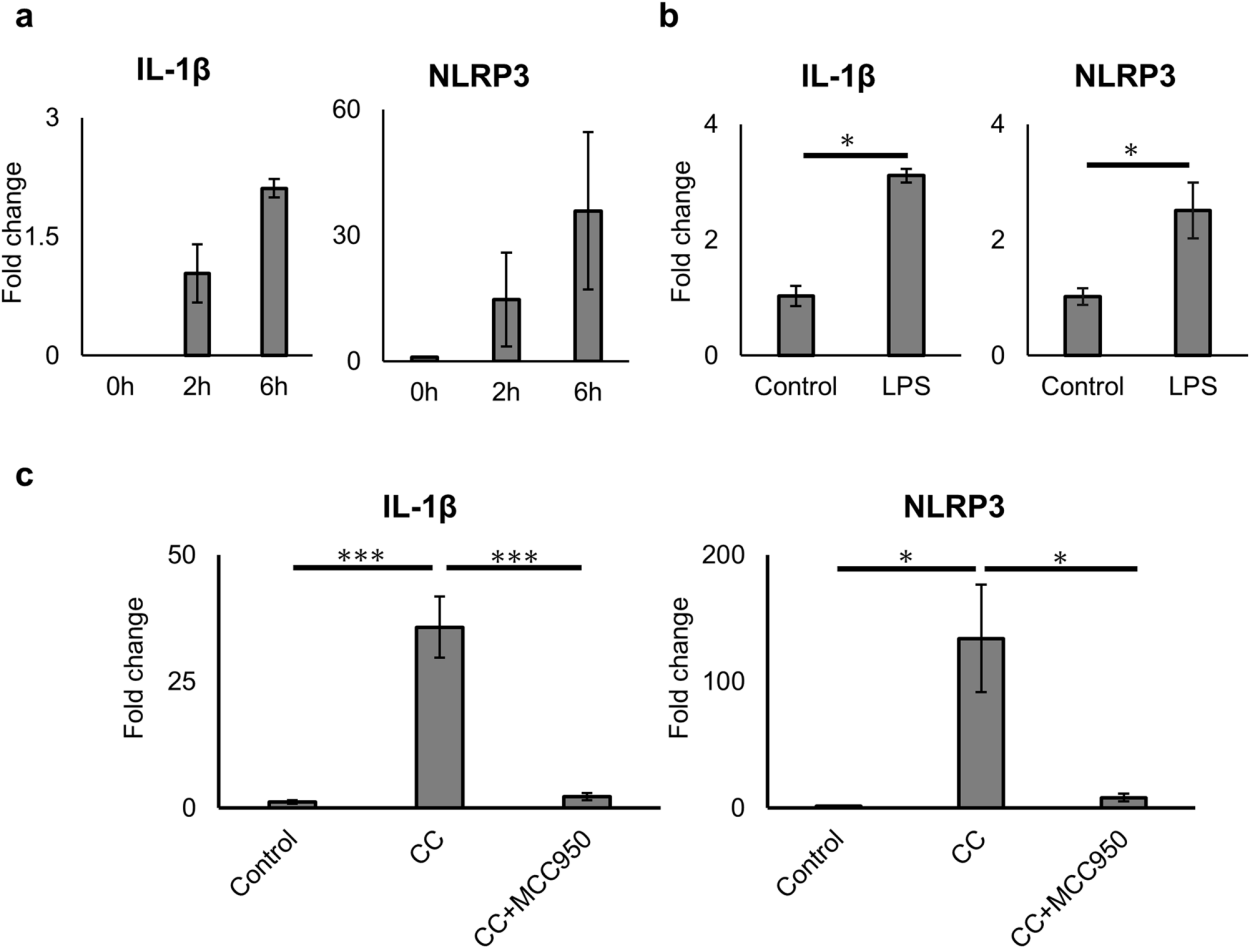
Upregulation of NLRP3 inflammasome-related genes in cultured islets. (**a**) Gene expressions of *Il1β* and *Nlrp3* in MIN6 incubated with a cytokine cocktail for 0, 2, and 6 h. (**b**) Gene expressions of *Il1β* and *Nlrp3* in isolated islets incubated with or without (control) lipopolysaccharide (LPS). (**c**) Isolated islets were incubated with a cytokine cocktail or MCC950. Gene expressions of *Il1β* and *Nlrp3* in isolated islets with cytokine stimulation (CC), CC and 10 µM of MCC950 (CC + MCC950), or no treatment (control). Data are shown as means ± standard error (SEM). Significant differences are indicated as *p < 0.05, **p < 0.01, ***p < 0.001.

First, we assessed the expression of NLRP3 inflammasome-related genes in MIN6 or islets stimulated by cytokines. We investigated the expression of *Il1b* and *Nlrp3* genes in MIN6 incubated with a cytokine cocktail (CC) comprising TNF-α, IFN-γ, and IL-1β, which are induced in the early phase of islet transplantation. *Il1b* and *Nlrp3* were upregulated at 2 and 6 h after incubation time-dependently (Fig. 1a). Next, expression of NLRP3 inflammasome-related genes was evaluated in islets incubated with lipopolysaccharide (LPS), a major cytokine stimulating NLRP3 inflammasome factor that signals through TLR4. Similar to MIN6, gene expression was upregulated by LPS stimulation (Fig. 1b). The upregulation of *Il1b* and *Nlrp3* was also seen in islets stimulated with CC, similar to LPS stimulation. The expression of *Il1b* and *Nlrp3* genes was higher after cytokine stimulation compared with no cytokine stimulation (29.1 ± 14.1 fold, *Il1b*, *p* < 0.001, and 107 ± 77.1 fold, *Nlrp3*, *p* < 0.001, Fig. 1c). Furthermore, the upregulated expressions of both genes were inhibited by MCC950 treatment (*p* < 0.001, *Il1b* and *p* < 0.05, *Nlrp3*, Fig. 1c). These results suggested that inflammatory cytokine exposure induced the upregulation of NLRP3 inflammasome-related genes, which were suppressed by MCC950, although increase of caspase-1 activity in the islets with cytokine exposure was not seen by luciferase chemiluminescence assay (supplemental Fig. 1).

### Inhibition of NLRP3 inflammasome improves the islet survival rate and cell viability

**Figure 2:**
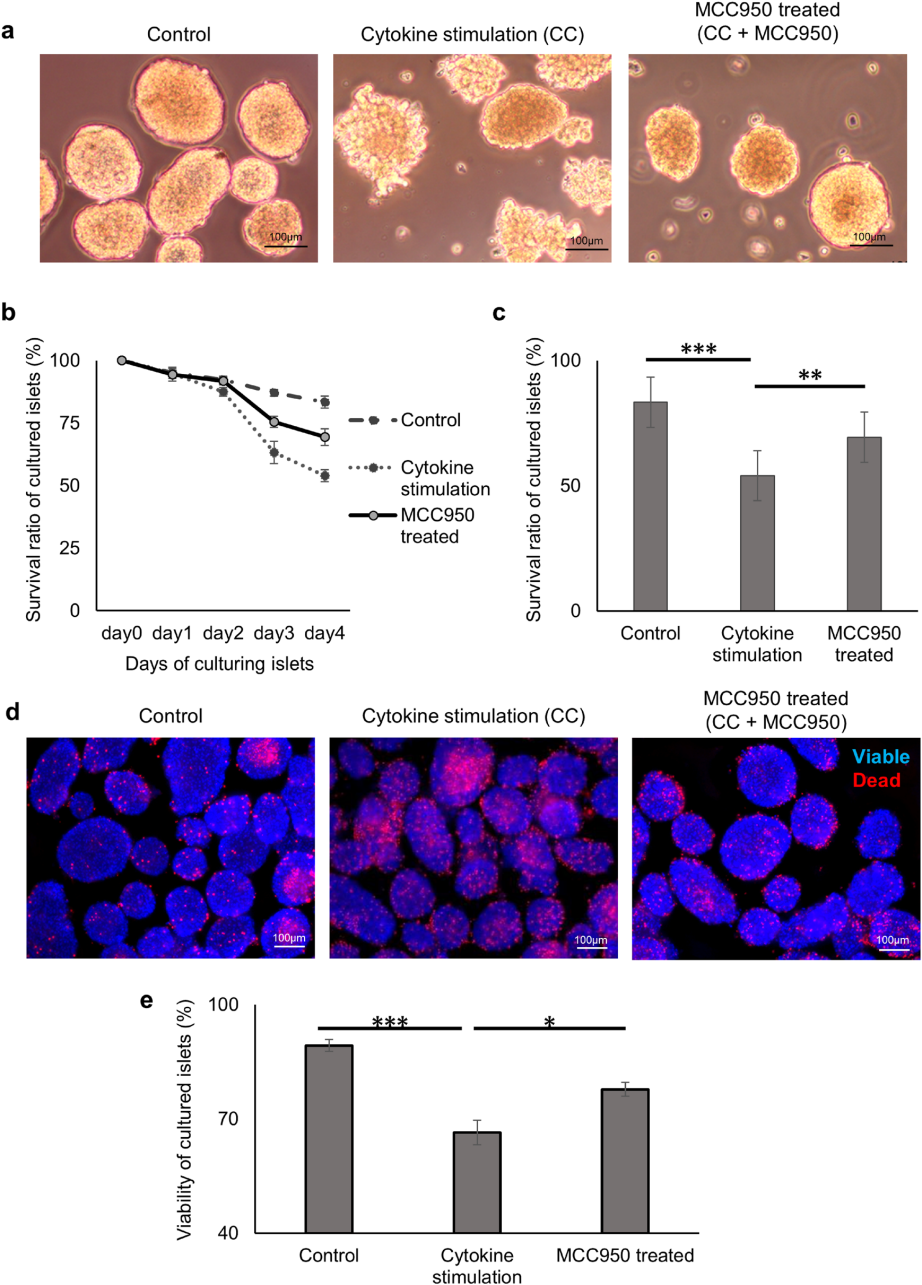
Inhibition of NLRP3 inflammasome improves the islet survival rate and cell viability under cytokine cocktail stimulation. (**a**) Morphological changes of islets cultured with the cytokine cocktail (CC), CC and MCC950 (CC + MC950), or no treatment (control) for 4 days. (**b**) The change in survival rate of cultured islets in CC, CC + MCC950, and control groups for 4 days (n = 5 for each group). (**c**) Survival rate of cultured islets at 4 days. (**d**) Hoechst/propidium iodide (PI)-stained islets at 24 h in CC, CC + MCC950, and control groups. Blue: viable, red: dead. (**e**) Viability of cultured islets at 24 h in CC, CC + MCC950, and control groups (n = 9 for each group).

Next, the number of surviving islets cultured with the CC, CC and MCC950 (CC + MCC950), or no treatment (control) were measured for 4 days to identify the impact of cytokine exposure on cultured islets and the effect of MCC950 on cultured islets. In the CC group, cultured islets had an irregular surface and some islets were destructively dispersed, whereas the morphology of islets was preserved (round-shape) in the control group. Of note, MCC950 protected the morphology of islets stimulated by cytokines (Fig. 2a). The survival rate of cultured islets in the CC group was significantly decreased to 53.8% on day 4 compared with 83.8% in the control group, and MCC950 significantly recovered the survival rate of islets (69%, one-way ANOVA; *p* < 0.001, Control vs CC; *p* < 0.0001, MCC950 vs CC; *p* = 0.0051. Figure 2b and c). Additionally, cell viability was calculated by Hoechst/PI staining to identify cellular death at 24 h, which reflected the survival rate of islets. The number of PI stained dead cells was increased in cytokine stimulated islets, whereas MCC950 reduced the number of PI stained cells (Fig. 2d). The cell viability of islets after 24 h of culture was reduced by cytokine stimulation, but significantly improved by MCC950 (Control vs CC vs CC + MCC950 treated group; 89.3% vs 66.4% vs 77.8%, one-way ANOVA; *p* < 0.001. Control vs CC; *p* < 0.001, Control vs MCC950; *p* = 0.0081, CC vs MCC950; *p* = 0.009, Fig. 2e). These results indicated NLRP3 inflammasome induced the cellular death of islets after cytokine exposure, and that MCC950 improved islet survival by inhibiting NLRP3 inflammasome.

### Apoptosis is induced in islets after transplantation and attenuated by MCC950 administration

**Figure 3:**
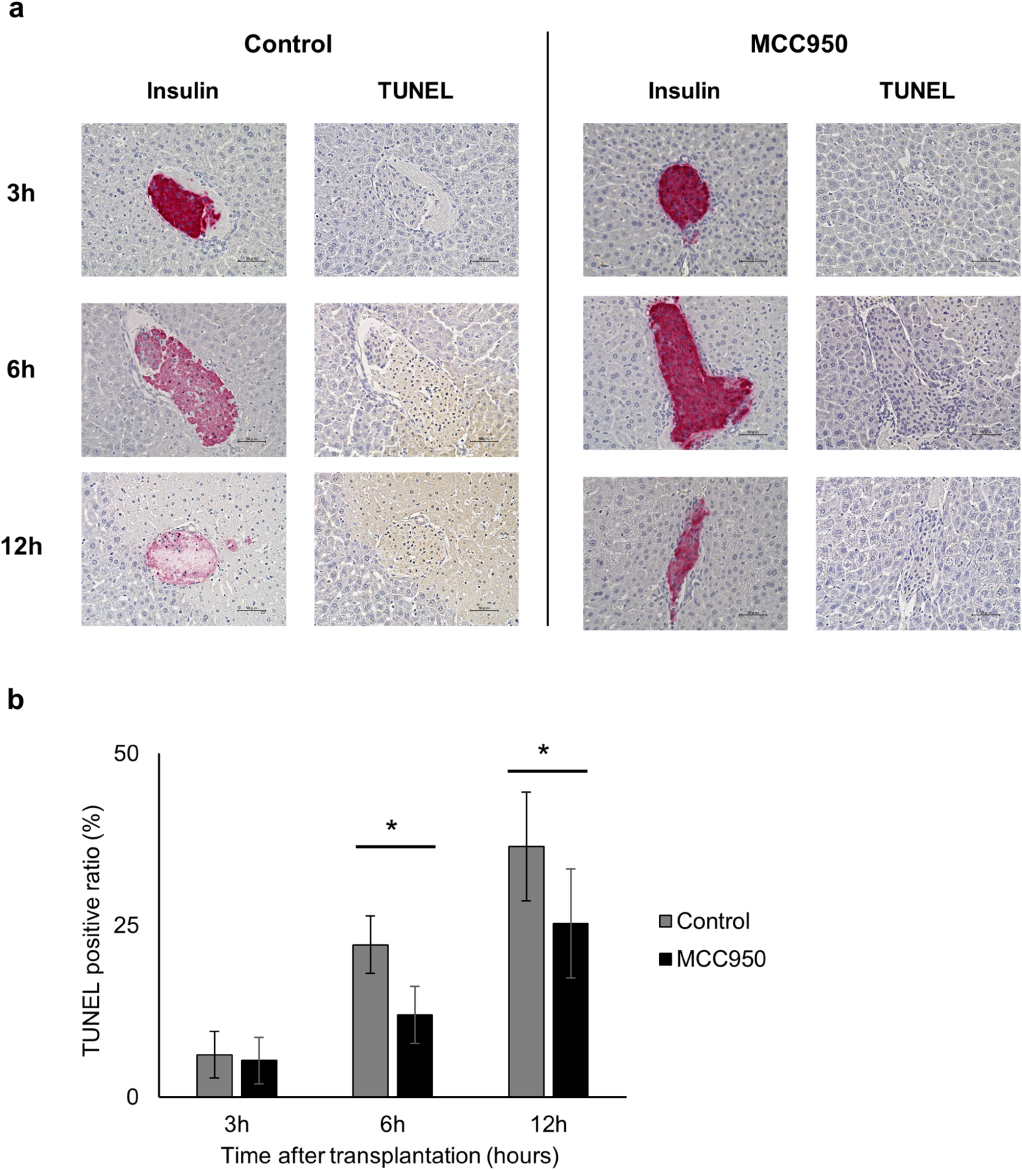
Expression of terminal deoxynucleotidyl transferase dUTP nick end labelling (TUNEL) positive cells in transplanted islets. (**a**) Diabetic recipient mice treated with or without MCC950 were transplanted with syngeneic islets (MCC950 or control group, respectively). Livers were recovered at 3, 6, and 12 h after transplantation. Sections were stained for insulin (red: left) and TUNEL-positive apoptotic cells (brown: right). (**b**) The ratio of TUNEL positive cells in the islet area at 3, 6, and 12 h after transplantation in MCC950 (black) and control (grey) groups. Data are shown as means ± SEM. Statistics were performed by the Student’s *t*-test. Significant differences are indicated as *p < 0.05.

The apoptosis of transplanted islets in the early phase of transplantation was assessed to elucidate the cellular death of islets in the liver. Terminal deoxynucleotidyl transferase dUTP nick end labelling (TUNEL) staining revealed that numbers of apoptotic islet cells were increased time-dependently at 3, 6, and 12 h after transplantation in the control group. The apoptosis of transplanted islets peaked at 6 h after transplantation. However, in the MCC950 treated group, apoptosis in islet cells was significantly reduced compared with the control group (Fig. 3a). The calculated ratio of TUNEL positive cells in transplanted islets (TUNEL positive ratio) was significantly recovered by MCC950 administration (control vs MCC950; 22.2 ± 8.6% vs 12.0 ± 4.2% at 6 h; *p* = 0.0218, and 36.5 ± 12.9% vs 25.3 ± 7.9% at 12 h; *p* = 0.0015. Figure 3b). This indicated that NLRP3 inflammasome was associated with the apoptosis of transplanted islets and that MCC950 suppressed apoptosis.

### Expression of IL-1β in the islet transplanted site is inhibited by MCC950 administration

**Figure 4:**
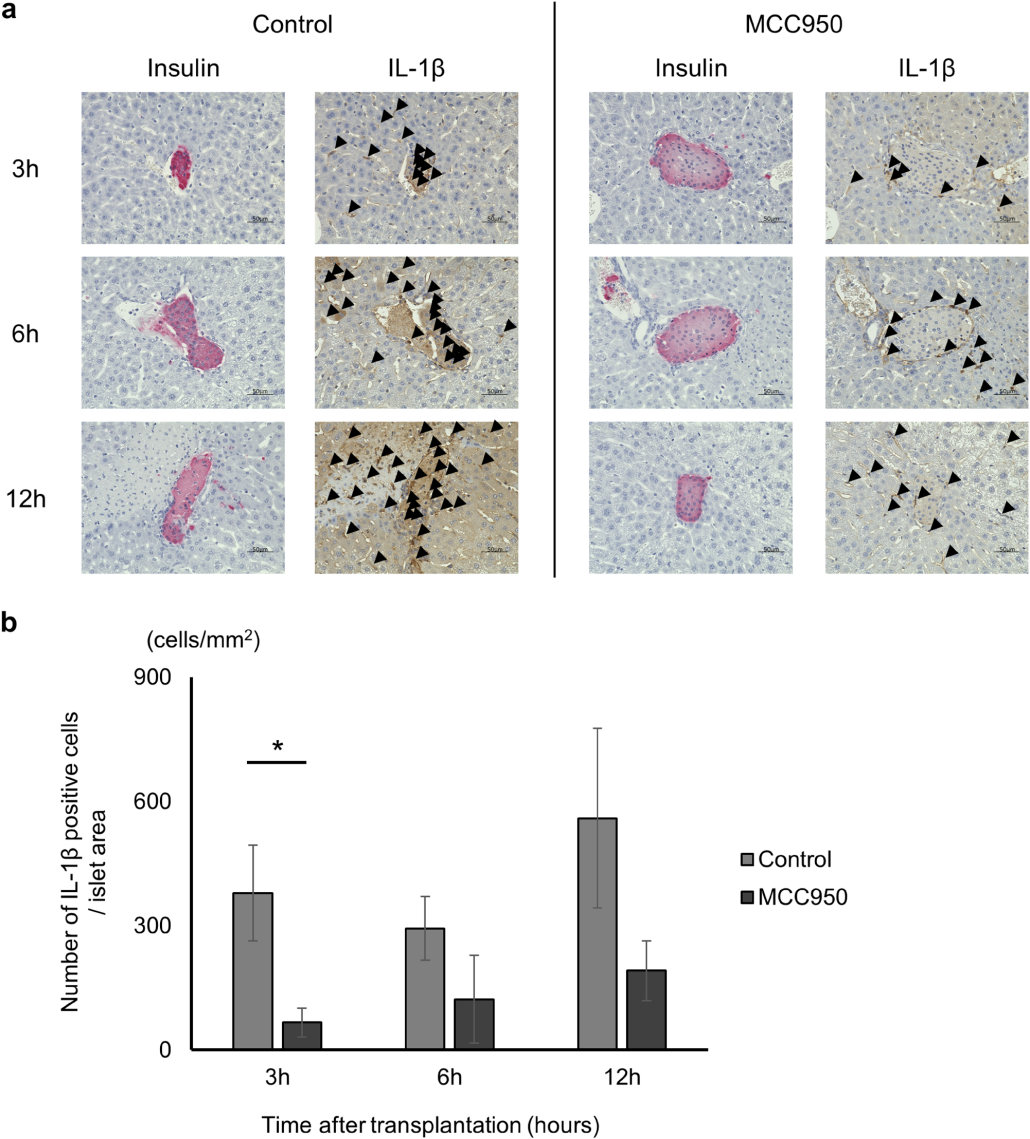
Expression of IL-1β in transplanted sites. (**a**) Specimens of transplanted islets at 3, 6, and 12 h after transplantation stained for insulin (green), IL-1β (red), and nuclei (blue). (**b**) The number of IL-1β positive cells/islet area in MCC950 (black) and control (grey) groups. Data are shown as means ± SEM. Significant differences are indicated as *p < 0.05.

To evaluate whether IL-1β produced by NLRP3 inflammasome was associated with islet transplantation, immunohistochemical staining for IL-1β in transplanted islet specimens recovered at 3, 6, and 12 h after transplantation was performed. Histopathological findings revealed IL-1β expression in islets and around islets in the early phase of transplantation. In the control group, IL-1β-positive cells, which was pointed by black arrows, in and around transplanted islets were detected at 3 h after transplantation and the numbers increased time-dependently (Fig. 4a and b). However, MCC950 treatment significantly reduced the number of IL-1β-positive cells in and around transplanted islets at 3 h after transplantation compared with the control group (Control group vs MCC950 group; 378.8 ± 116.0 cells/mm^2^ vs 66.0 ± 35.0 cells/mm^2^ at 3 h; *p* = 0.0043, 293.0 ± 77.0 cells/mm^2^ vs 121.9 ± 105.9 cells/mm^2^ at 6 h; *p* = 0.1226, and 559.1 ± 216.5 cells/mm^2^ vs 191.2 ± 72.1 cells/mm^2^ at 12 h; *p* = 0.1122, Fig. 4b). These results suggested that NLRP3 inflammasome induced IL-1β in transplanted islets, which was prevented by MCC950.

### Macrophage migration around transplanted islets is attenuated by MCC950

**Figure 5:**
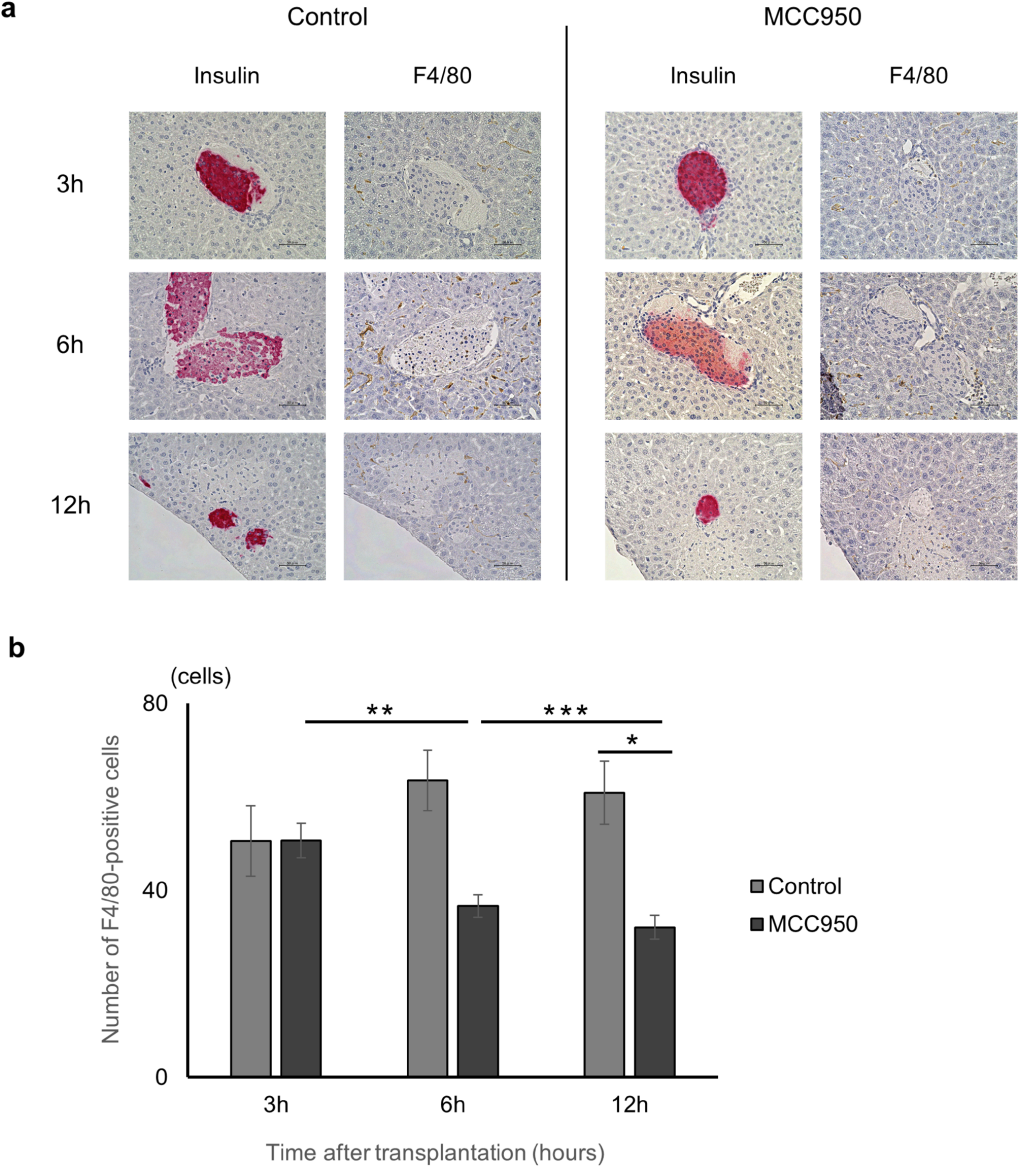
Expression of F4/80 positive cells. (**a**) Specimens of transplanted islets at 3, 6, and 12 h after transplantation stained for insulin (red) and F4/80 (brown). (**b**) The number of F4/80 positive cells around transplanted islets in MCC950 (black) and control (grey) groups. Data are shown as means ± SEM. Significant differences are indicated as *p < 0.05, **p < 0.01, ***p < 0.001.

To evaluate the number of macrophages around transplanted islets, liver samples were stained for F4/80 at 3, 6, and 12 h after transplantation. In the control group, numbers of F4/80^+^ macrophages observed mostly in the sinusoids of the liver around transplanted islets gradually increased time-dependently (Fig. 5a). In the MCC950 treated group, F4/80^+^ macrophages were detected in the sinusoids of the liver around islets at 3 h after transplantation similar to the control group. However, numbers of F4/80^+^ macrophages were reduced in the MCC950 group at 6 and 12 h after transplantation compared with the control group (Control vs MCC950 group; 50.6 ± 7.5 cells vs 50.7 ± 3.7 cells at 3 h; *p* = 0.5067, 63.5 ± 6.5 cells vs 36.6 ± 2.4 cells at 6 h; *p* = 0.0037, and 60.9 ± 6.7 cells vs 32.1 ± 2.6 cells at 12 h; *p* = 0.0015. Figure 5b). Therefore, MCC950 attenuated the migration of macrophages around the transplanted islets by inhibiting NLRP3 inflammasome in the early phase after transplantation.

### MCC950 improves glycaemic control in diabetic mice after syngeneic islet transplantation

**Figure 6:**
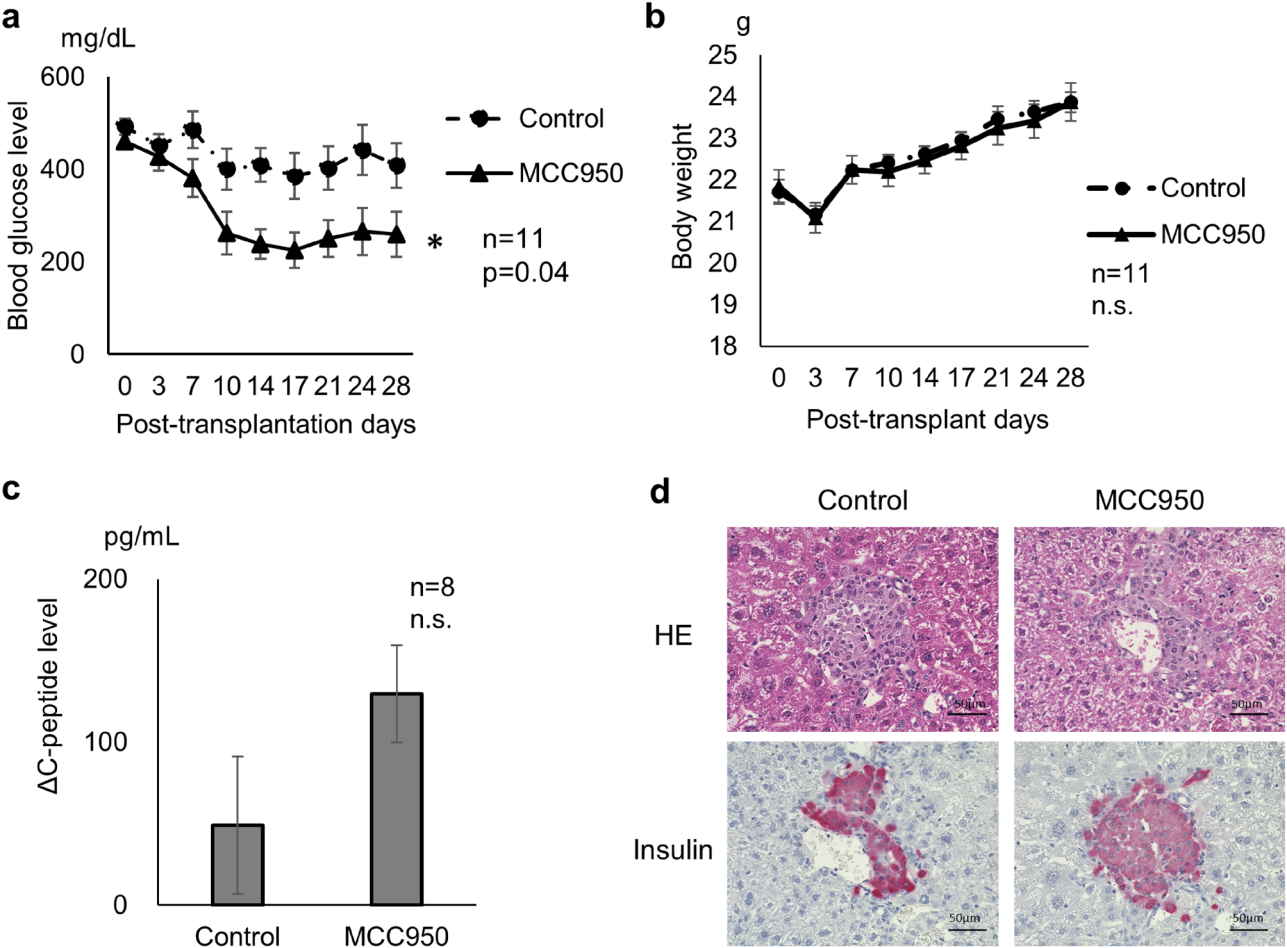
Effect of an NLRP3 inflammasome inhibitor on islet transplantation. (**a**) Blood glucose profiles in MCC950 and control groups 28 days after transplantation. (**b**) Body weight changing was demonstrated for 28 days after transplantation. (**c**) ΔC-peptide level by glucose stimulation was demonstrated as a bar graph. (**d**) Histological examination at 30 days after transplantation to detect engrafted islets. Data are shown as means ± SEM. Significant differences are indicated as *p < 0.05.

Finally, the therapeutic effect of islet transplantation was compared between MCC950 treated and control groups. The blood glucose level was measured for 28 days after islet transplantation. Blood glucose levels were significantly lower in the MCC950 group compared with the control group (repeated measures ANOVA; *p* = 0.0401, Fig. 6a). On the other hand, body weight changing after islet transplantation was demonstrated as almost similar trend in both control and MCC treated group (Fig. 6b). MCC950 might have another effect indirectly to affect appetite. Regarding plasma C-peptide level, MCC950 treated group had increased ΔC-peptide level in response to glucose stimulation (Fig. 6c). After 1 month, livers were removed to assess the histology of the islet engraftment. Engraftment of transplanted islets was detected in both control and MCC950 groups (Fig. 6d).

## Discussion

This study investigated the impact of NLRP3 inflammasome on islet transplantation. NLRP3 inflammasome is an intracellular sensor and its assembly leads to the caspase-1 dependent release of the proinflammatory cytokines, IL-1β and IL-18^17^. Additionally, NLRP3 inflammasome induces pyroptosis, an apoptosis pathway that leads to cellular death. We found that cytokine stimulation promoted NLRP3 inflammasome-related gene expression and impaired islet cell viability. We used the cytokine stimulation model to reflect inflammatory conditions during intrahepatic islet transplantation, which can impair islet engraftment. We also showed the therapeutic effects of MCC950, a small molecule that inhibits NLRP3 inflammasome, which was developed based on diarylsulfonylurea-containing compounds that are novel IL-1β processing inhibitors^18,19^. MCC950 administration suppressed NLRP3 inflammasome-related gene expression, improved islet viability, prevented the apoptosis of transplanted islets, reduced the production of IL-1β in transplanted islets and migration of macrophages, and improved the glycaemic control of islets after transplantation. Therefore, MCC950 might be a new therapeutic agent to improve islet transplantation.

The inflammatory process of NLRP3 inflammasome was characterized as a two-step process including priming and activation^17^. Priming of NLRP3 inflammasome is initiated to upregulate the expression of inflammasome components. This upregulation is induced by the recognition of pathogen-associated molecular patterns (PAMPs), damage-associated molecular patterns (DAMPs), ATP, and cytokines. The second process involves activation and inflammasome formation^17,20^. NLRP3 inflammasome is activated by the recognition of NLRP3 activators, such as DAMPs, reactive oxygen species (ROS), oxidant stress, and mitochondrial dysfunction. The activated inflammasome cleaves caspase-1 and activates pro-IL-1β to IL-1β. The current study found that TNF-α, IFN-γ, and IL-1β upregulated NLRP3 inflammasome-related gene expression in mouse islets. Furthermore, inhibition of NLRP3 inflammasome by MCC950 prevented the upregulation of these genes induced by proinflammatory cytokines. In the transplant model, histological analysis indicated IL-1β was produced in transplanted islets from early after transplantation, and was reduced by MCC950 especially at 3 h after transplantation. Moreover, high numbers of F4/80^+^ macrophages that migrated around transplanted islets and within hepatic parenchyma near the islets were reduced by MCC950. These therapeutic effects on macrophages were seen after 6 h, which was delayed compared with the suppressive effect on IL-1β production in islets. These results suggested that chemically and/or mechanically damaged islets released DAMPs and proinflammatory cytokines including IL-β, which triggered subsequent inflammatory reactions. Although the amount of IL-1β produced by islets was low compared with immune cells, it promoted inflammation during islet transplantation. The suppression of IL-1β production in islets reduced the migration of macrophages to the liver. Previous reports revealed that IL-1β was produced in human islets through NLRP3 inflammasome^7^ and that NLRP3 inflammasome was activated in rat islets by hypoxia^21^. And IL-1β has known to lead to Fas receptor upregulation, NF-κB activation, β cell apoptosis, and dysfunction^22^. Our results suggested that NLRP3 inflammasome regulated IL-1β production in mouse islets and macrophages, and that MCC950 suppressed IL-1β production in transplanted islets and existing macrophages, and reduced the migration of macrophages around transplanted islets. These suppressive effects of IL-1β might contribute to attenuated glucose fluctuations after islet transplantation.

NLRP3 inflammasome induces pyroptosis, a form of apoptosis. Recent studies reported the N-domain of gasdermin D (GSDMD) mediated proinflammatory pyroptosis^23–25^. GSDMD has two domains (N-domain and C-domain). Activated caspase-1 and caspase-11 cleave GSDMD into two fragments. The N-domain of GSDMD forms pores on lipid membranes and induces pyroptosis through cell membrane disruption. Our study revealed that induction of NLRP3 inflammasome by cytokine stimulation or islet transplantation caused islet cellular apoptosis (pyroptosis), and that inhibition by MCC950 improved the cellular survival of islets in vitro and in vivo. Islets were damaged by chemical and mechanical manipulation during the isolation process, which induced apoptosis. Regarding NLRP3 associated apoptosis, Wang et al. reported that islet β cell apoptosis was mediated by NLRP3 inflammasome through the elevation of serum angiotensin II^26^, whereas the Lebreton group reported NLRP3 inflammasome activation was not involved in islet cell death induced by hypoxia^7^. In our mouse islet transplant model, MCC950 restored islet damage induced during the isolation process.

IL-1β is an important inflammatory mediator responsible for islet dysfunction^5,27,28^. Therefore, the regulation of IL-1β is important to prevent early rejection and improve the therapeutic outcome of islet transplantation. Nazirrudin et al. reported an improved outcome for clinical islet transplantation by the combined blockade of IL-1β and TNF-α using anakinra and etanercept^29^. Additionally, IL-1β upregulated CXCL10 in islets, and the inhibition of CXCL10 improved the outcome of islet transplantation. MCC950 blocks NLRP3 inflammasome, which suppresses upstream IL-1β production. The effects of MCC950 were recently reported for metabolic diseases such as NASH. Auvro et al. showed that NLRP3 inflammasome blockade by MCC950 reduced the number of macrophages in the livers of NASH mice^30^. Likewise, our study revealed that NLRP3 inflammasome blockade by MCC950 reduced the number of macrophages in livers during islet transplantation and that MCC950 improved the engraftment of islets by reducing apoptosis (pyroptosis). On the other hand, the role of IL-1β to β cell has paradoxical features regarding glucose metabolism associated with inflammatory cells as homeostasis, and IL-1β blockade could potentially affect β-cell physiological activity which was independent of engraftment after transplantation. While IL-1β promotes postprandial insulin secretion^31^ and short-term exposure to macrophage-derived IL-1β stimulate insulin secretion, Donath et.al. described that long-term may exposure to IL-1β may lead to β-cell exhaustion^32^. As the limitation of this study, we did not identify caspase-1 activity in islets under cytokine stimulation in in vitro assay shown in Supplemental Fig. 1, despite upregulation of NLRP3 related inflammasome in the islets and increase of IL-1β positive cells after islet transplantation were seen. This discrepancy suggests that, in vivo condition, transplanted islets were affected by not only cytokine stimulation but also other factors which is still unknown. And to completely pass thorough the NLRP3 inflammasome pathway including caspase-1 activation, this unknown factor might be required. To clarify this unknown factor, future studies should clarify the role of NLRP3 inflammasome in islet transplantation and the detailed mechanism of pyroptosis inhibition by MCC950.

In conclusion, we revealed that NLRP3 inflammasome worsened the therapeutic outcomes of islet transplantation. Furthermore, MCC950 administration improved glycaemic control in a syngeneic mouse islet transplantation model by suppressing islet death, the production of IL-1β in islets, and migration of macrophages.

## Materials and methods

### Animals

Nine-week-old male C57BL/6 J mice, purchased from CLEA Japan Inc. (Tokyo, Japan), were used as diabetic recipients and donors for islet transplantation. All mice were bred under specific pathogen-free conditions. This study was approved by the Fukuoka University Animal Care and Use Committee (ID: 1,808,052) and carried out under the Fukuoka University Animal Experimentation Regulations.

### Islet isolation

Pancreata were expanded by an injection of collagenase solution (collagenase type V, Sigma-Aldrich, St. Louis, USA) into the pancreatic duct under laparotomy with general anaesthesia using isoflurane (Fujifilm Wako Pure Chemical Co., Osaka, Japan). Expanded pancreata were digested by incubating at 37 °C for 18 min with shaking. Digested tissue was washed with Hanks’ balanced salt solution (HBSS, Gibco-Thermo Fisher Scientific, Waltham, MA, USA), and islets were purified by discontinuous density gradient centrifugation using Biocoll (1.077 and 1.100 g/ml, Biochrom, Berlin, Germany). After collection of purified islets, they were handpicked under a microscope for additional purification. Isolated islets were used in subsequent experiments after overnight culturing in Dulbecco’s modified Eagle’s medium (DMEM, Thermo Fisher Scientific, Waltham, MA, USA) supplemented of 10% foetal bovine serum (Thermo Fisher Scientific) and 1% Penicillin–Streptomycin (Thermo Fisher Scientific) at 37 °C in 5% CO_2_.

### Cell culture

The MIN6, β cell line, was cultured in Dulbecco’s modified Eagle medium (DMEM) containing 4,500 mg/dL glucose, supplemented with 10% foetal bovine serum and 1% antibiotics in 5% CO_2_ at 37°C^33^. The cells were used for subsequent RT-PCR after reaching 70% confluence.

### Quantitative RT-PCR

MIN6 cells or islets were incubated with a cytokine cocktail (DMEM containing 5 ng/mL IL-1β (Fujifilm Wako Pure Chemical Co.), 10 ng/mL TNF-α (Fujifilm Wako Pure Chemical Co.) and 50 ng/mL IFN-γ (Fujifilm Wako Pure Chemical Co.) for 4 h to induce cytokine production within islets (defined as “CC”). The groups incubated with MCC950 (10 µM) or not treated were defined as “CC + MCC950” and “control”, respectively. After treatments, MIN6 cells or islets were lysed with TRIzol Reagent (Thermo Fisher Scientific) and RNA was extracted from lysates using the PureLink RNA Mini Kit (Thermo Fisher Scientific). RNA was reverse transcribed using the High-Capacity cDNA Reverse Transcription Kit (Thermo Fisher Scientific). Quantitative real-time PCR for cDNA was performed using the LightCycler96 System (Roche) with TB Green *Premix Ex Ta* II (Takara Bio Inc., Shiga, Japan) for amplification and detection. The expressions of *Il1b* and *Nlrp3* were normalized to the expression of *Rplp0*. The primers were as follows: 5′-TCCAGGATGAGGACATGAGCAC-3′ (forward) and 5′-GAACGTCACACACCAGCAGGTTA-3′ (reverse) for *Il1b*, 5′-AATGCCCTTGGAGACACAGGA-3′ (forward) and 5′-TGAGGTGAGGCTGCAGTTGTCTA-3′ (reverse) for *Nlrp3*, 5′-GGCAGCATTTATAACCCTGAAGTG-3′ (forward) and 5′-TGTACCCATTGATGATGGAGTGTG-3′ (reverse) for *Rplp0*.

### Islet survival assay

Fifty islets were cultured in 2 mL of DMEM with the cytokine cocktail (CC), the cytokine cocktail and 10 µM MCC950 (CC + MCC950), or were untreated (control), at 37 °C in 5% CO_2_ for 4 days. The survival ratio of cultured islets was defined as the number of islets/the number of islets at preincubation and was measured every day.

### Cell viability of islets assay

Isolated islets were incubated with the cytokine cocktail containing-media for 24 h in the presence of 0 and 10 µM MCC950. Control islets were untreated with cytokine cocktail and MCC950. Then, islets were stained with propidium iodide (PI, Sigma-Aldrich) to detect dead cells and Hoechst 33,342 (Sigma-Aldrich) to detect viable cells^34^. We obtained images with a fluorescence microscope (BZ-X710, Keyence, Osaka, Japan). The viability of cultured islets was determined as follows: viability of cultured islets (%) = (number of viable cells/number of total cells) × 100.

### Islet transplantation

Diabetes was induced by streptozotocin (STZ; 180 mg/kg mouse body weight, Sigma-Aldrich) injection into the tail vein of C57BL/6 J mice. Diabetic mice with a blood glucose level of 400–600 mg/dL for two consecutive days at 3–5 days after the administration of streptozotocin were used as recipients. For transplantation, 170 islet equivalents (IEQs) were transplanted into the liver via the portal vein. The treatment group (defined as the MCC950 group), was intraperitoneally (ip) injected with 25 mg/kg MCC950 at 30 min before and 6, 24, and 48 h after transplantation. For the control group, phosphate buffered saline (PBS) was injected at the same time points as the MCC950 group. Non-fasting blood glucose levels and body weight were monitored in all recipients twice a week until 28 days after islet transplantation. After 20 days after transplant for each 8 mice per group, plasma C-peptide level was measured by enzyme-linked immunosorbent assay (ELISA). Blood samples as pre-stimulation were collected from tail vein after 12–14 h fasting. And 20% glucose solution was intraperitoneally injected at dose of 2 g / kg of mice body weight. Blood samples as post-stimulation were collected at 30 min after glucose injection. Plasma samples were obtained by centrifugation at 3,000 rpm for 5 min, and plasma C-peptide level was measured using LEBIS mouse C-peptide ELISA kit, U type (FUJIFILM Wako Shibayagi Corporation, Shibkawa, Japan). ΔC-peptide was calculated as plasma C-peptide level of post-stimulation subtracted by plasma C-peptide level of pre-stimulation (pg/mL).

### Histopathology and immunohistochemistry

The liver was removed at 3, 6, or 12 h, or 30 days after transplantation under general anaesthesia and fixed in 10% formaldehyde solution. The samples were embedded in paraffin, cut into 3-μm-thick sections, deparaffinized in xylene, and rehydrated in ethanol. The sections were stained with haematoxylin and eosin (HE), and immunohistochemical staining was performed to detect islets (insulin), IL-1β, macrophages (F4/80), or apoptotic cells using TUNEL. The primary antibodies for immunohistochemistry were rabbit monoclonal anti-insulin antibody (diluted 1:5000; ab181547, Abcam plc, Cambridge, UK), polyclonal goat IgG anti-mouse IL-1β/IL-1F2 antibody (diluted 1:25; R&D Systems Inc), and rabbit monoclonal anti-F4/80 antibody (diluted 1:250; #70,076, Cell Signaling Technology, Danvers, MA, USA). After incubation of the primary antibody, donkey anti-rabbit secondary antibody (diluted 1:500; ab97082 or ab6802, Abcam plc) or donkey anti-goat IgG H&L (HRP polymer, for IL-1β staining; Abcam) was applied. To detect the target cells, a Warp Red Chromogen Kit (for insulin; Biocare Medical, Pacheco, CA, USA BRR806AH) and diaminobenzidine (DAB; for IL-1β and F4/80) were used. Regarding TUNEL staining, a TdT In Situ Apoptosis Detection Kit (R&D Systems, Inc, Minneapolis, MN USA 481,030-k) was used following the manufacturer’s instructions. Images were captured using a BZ-X710 microscope and imaging analysis was performed using ImageJ software (National Institute of Mental Health, Bethesda, Maryland, USA).

### Statistical analysis

Data are shown as the means ± standard error of the mean (SEM). Differences were assessed by Welch’s test for parametric data with unequal variances or Wilcoxon rank sum test for non-parametric data between two groups, one-way ANOVA for data among three groups, or repeated measurement ANOVA for the in vitro survival rate and blood glucose trend after transplantation. When one-way ANOVA was applied, the Tukey–Kramer HSD test was used to identify significant differences between groups. Significant differences were determined when *p* < 0.05.

## Supplementary information

Supplementary Information
