# Supplementary material for: Inhibition of NLRP3 inflammasome by MCC950 improves the metabolic outcome of islet transplantation by suppressing IL-1β and islet cellular death
Source: Sci Rep. 2020 Oct 21;10:17920. doi: 10.1038/s41598-020-74786-3 (PMC7578017; doi:10.1038/s41598-020-74786-3)

Inhibition of NLRP3 inflammasome by MCC950 improves the metabolic outcome of islet transplantation by suppressing IL-1β and islet cellular death

Taisuke Matsuoka[1], Gumpei Yoshimatsu[1*], Naoaki Sakata[1], Ryo Kawakami[1], Tomoko Tanaka[1], Teppei Yamada[2], Yoichiro Yoshida[2], Suguru Hasegawa[2], Shohta Kodama[1]

1. Department of Regenerative Medicine and Transplantation, Fukuoka University, Fukuoka, Japan
2. Department of Gastroenterological surgery, Fukuoka University, Fukuoka, Japan

Supplemental data

Materials and Method

Assessment of caspase-1 activity on islets using luciferase assay system

Isolated islets were incubated for 0, 3, 6, 12, 24 hours in RPMI based culture medium with cytokine cocktail (TNF α, IFN γ, IL-1 β; CC group), without cytokine cocktail (Control group) or cytokine cocktail and MCC950 (CC + MCC950 group). After that, cellular caspase-1 activity on the islets was assessed using Caspase-Glo 1 inflammasome assay kit (Promega, Madison, WI, USA). Caspase-1 activity through chemiluminescence by luciferase was measured according to the manufacture-provided protocol. Chemiluminescence was measured by Spark (TECAN, Männedorf, Switzerland).

Result

Increase of caspase-1 activity in the islets with cytokine exposure was not seen by luciferase chemiluminescence assay (supplemental figure 1). As the limitation of this study, we could not identify caspase 1 activity in islets under cytokine stimulation in in vitro assay shown in Supplemental Figure 1, despite upregulation of NLRP3 related inflammasome in the islets and increase of IL-1β positive cells after islet transplantation were seen. This discrepancy suggests that, in vivo condition,

transplanted islets were affected by not only cytokine stimulation but also other factors which is still unknown. And to completely pass thorough the NLRP3 inflammasome pathway including caspase-1 activation, this unknown factor might be required. To clarify this unknown factor, future studies should clarify the role of NLRP3 inflammasome in islet transplantation and the detailed mechanism of pyroptosis inhibition by MCC950.

Figure legend

Supplemental Figure 1. Caspase-1 activity of islets.

Isolated islets were incubated for 0, 3, 6, 12, 24 hours in RPMI based media as Control group, in the media with cytokine cocktail as CC group, and in the media with cytokine cocktail and MCC950 as CC + MCC950 group (n=4 per each group). Data are shown as means ± SEM.

Supplemental figure 1

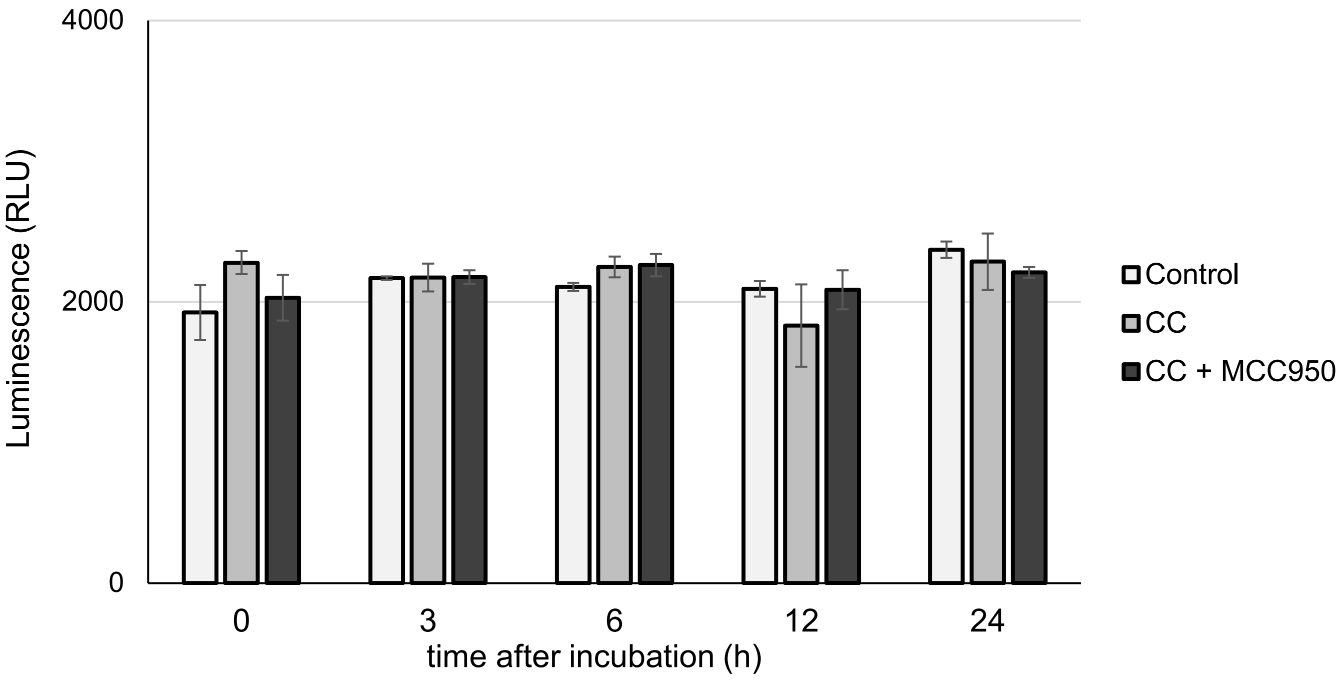

Supplement: Supplementary file 1 — Supplementary Information [file 41598_2020_74786_MOESM1_ESM.pdf]
